# Impact of Nuclear Export Pathway on Cytoplasmic HIV-1 RNA Transport Mechanism and Distribution

**DOI:** 10.1128/mBio.01578-20

**Published:** 2020-11-10

**Authors:** Jianbo Chen, Chijioke Umunnakwe, David Q. Sun, Olga A. Nikolaitchik, Vinay K. Pathak, Ben Berkhout, Atze T. Das, Wei-Shau Hu

**Affiliations:** a Viral Recombination Section, HIV Dynamics and Replication Progam, National Cancer Institute, Frederick, Maryland, USA; b Viral Mutation Section, HIV Dynamics and Replication Program, National Cancer Institute, Frederick, Maryland, USA; c Laboratory of Experimental Virology, Department of Medical Microbiology, Amsterdam University Medical Centers, Amsterdam, The Netherlands; Icahn School of Medicine at Mount Sinai; Albert Einstein College of Medicine

**Keywords:** diffusion, HIV-1, nuclear export, RNA, cytoplasmic, distribution, transport mechanism

## Abstract

HIV-1 full-length RNA (referred to as HIV-1 RNA here) serves as the viral genome in virions and as a template for Gag/Gag-Pol translation. We previously showed that HIV-1 RNA, which is exported via the CRM1 pathway, travels in the cytoplasm mainly through diffusion. A recent report suggested that the export pathway used by retroviral RNA could affect its cytoplasmic transport mechanism and localization. HIV-1 RNA export is directed by the viral protein Rev and the *cis*-acting element, Rev response element (RRE). When Rev/RRE is replaced with the constitutive transport element (CTE) from Mason-Pfizer monkey virus (MPMV), HIV-1 RNA is exported through the NXF1 pathway. To determine the effects of the export pathway on HIV-1 RNA, we tracked individual RNAs and found that the vast majority of cytoplasmic HIV-1 RNAs travel by diffusion regardless of the export pathway. However, CTE-containing HIV-1 RNA diffuses at a rate slower than that of RRE-containing HIV-1 RNA. Using *in situ* hybridization, we analyzed the subcellular localizations of HIV-1 RNAs in cells expressing a CTE-containing and an RRE-containing provirus. We found that these two types of HIV-1 RNAs have similar subcellular distributions. HIV-1 RNA exported through the NXF1 pathway was suggested to cluster near centrosomes. To investigate this possibility, we measured the distances between individual RNAs to the centrosomes and found that HIV-1 RNAs exported through different pathways do not exhibit significantly different distances to centrosomes. Therefore, HIV-1 RNAs exported through CRM1 and NXF1 pathways use the same RNA transport mechanism and exhibit similar cytoplasmic distributions.

## Abstract (precis)

The unspliced HIV-1 full-length RNA (HIV-1 RNA) is packaged into virions as the genome and is translated to generate viral structural proteins and enzymes. To serve these functions, HIV-1 RNA must be exported from the nucleus to the cytoplasm. It was recently suggested that export pathways used by HIV-1 RNA could affect its cytoplasmic transport mechanisms and distribution. In the current report, we examined the HIV-1 RNA transport mechanism by following the movement of individual RNAs and identifying the distribution of RNA using *in situ* hybridization. Our results showed that whether exported by the CRM1 or NXF1 pathway, HIV-1 RNAs mainly use diffusion for cytoplasmic travel. Furthermore, HIV-1 RNAs exported using the CRM1 or NXF1 pathway are well mixed in the cytoplasm and do not display export pathway-specific clustering near centrosomes. Thus, the export pathways used by HIV-1 RNAs do not alter the cytoplasmic transport mechanisms or distribution.

## INTRODUCTION

Retroviral DNA is integrated into the cellular genome to become a provirus and a permanent part of the cell (1). Using the promoter located in the 5′ long terminal repeat (LTR), RNA polymerase II transcribes the provirus and generates viral transcripts, some of which are spliced, while others remain full length. Splicing patterns differ in various retroviruses. Some retroviruses, such as murine leukemia virus, generate one spliced RNA and the unspliced full-length RNA. Other retroviruses, such as HIV-1, have complex splicing regulation that generates several types of completely, partially, and unspliced transcripts. In general, retroviruses use spliced transcripts to express viral proteins, and the unspliced full-length RNA serves as a template for Gag/Gag-Pol translation and as the viral genome that is packaged into virions (2). Thus, it is important for retroviruses to ensure that partially spliced and unspliced RNAs are exported to the cytoplasm.

Retroviruses use the host machinery for gene expression, including the nuclear export factors. Cellular mRNA export is closely coupled with RNA processing, including splicing, and most of the exported cellular mRNAs are spliced transcripts (3–6). The full-length viral RNA is unspliced and contains introns; thus, the virus must employ strategies to ensure that these RNAs are exported from the nucleus. HIV-1 and Mason-Pfizer monkey virus (MPMV) represent two of the well-defined examples of retroviruses utilizing different strategies to export their unspliced and partially spliced transcripts from nucleus to cytoplasm via distinct export pathways. HIV-1 uses a set of *cis*- and *trans*-acting elements to facilitate the export of partially spliced and unspliced RNA. HIV-1 encodes an accessory protein, Rev, that binds and multimerizes on a highly structured RNA element in the *env* gene, termed Rev response element (RRE) (7–13). Rev-RRE forms a complex with host export protein CRM1 (XpoI) and Ran-GTP, facilitating the export of the RNA-protein complex into the cytoplasm (14, 15). In contrast, the MPMV genome contains an RNA element, the constitutive transport element (CTE), which forms a complex with host export proteins NXF1/NXT1 to facilitate RNA export (16–18). Thus, different retroviruses use distinct export pathways to transport their intron-containing RNA to the cytoplasm. Interestingly, although the Rev/RRE and the CTE are distinct in their mechanisms of action, each system can functionally substitute for the other to support viral replication. Replacing Rev/RRE with CTE allows HIV-derived intron-containing viral RNA expression in a Rev- and CRM1-independent manner (16). Similarly, substituting the CTE with HIV-1 RRE, and supplementing Rev in *trans*, supports MPMV particle production (19). Interestingly, the strengths of these export elements may differ among retroviruses. It was shown that although the presence of one copy of MPMV CTE can mediate HIV-1 full-length RNA (HIV-1 RNA) export, the process is inefficient; however, the presence of four copies of MPMV CTE allows gene expression at levels similar to that from RRE-mediated RNA export (20). Hence, four copies of MPMV CTE are often used to replace RRE to achieve efficient RNA export (21, 22). There are multiple elements in the HIV-1 *gag-pol* that inhibit RNA export (23, 24); thus, it is possible that the Rev-RRE has evolved to overcome the effects of these inhibitory elements.

After exiting the nucleus, RNAs travel to various subcellular locations to serve their functions. The transport of cellular mRNA in the cytoplasm can be complex. Some mRNAs are transported in the cytoplasm predominantly by diffusion, whereas other mRNAs can be actively transported along the cytoskeleton by motor proteins to specific locations (25–30). By tracking movements of individual RNA molecules in living cells, we and others have shown that diffusion is the major transport mechanism used by unspliced HIV-1 RNA (31, 32). Furthermore, HIV-1 RNAs do not exhibit obvious clustering in a given subcellular location but are distributed throughout the cytoplasm (31). In this previous study, we used HIV-1 RNA containing authentic RRE; thus, these RNAs were exported via the CRM1-mediated pathway. In another recent study (33), it was shown that RNA containing mostly HIV-1 sequences along with RRE did not transport directionally and appeared to distribute throughout the cytoplasm, confirming our observations. Interestingly, this recent report also showed that when RRE was replaced with CTE from MPMV, the resulting RNA changed the transport mechanism and subcellular location (33). It was suggested that the NXF1 protein associated with CTE linked RNAs to the microtubules in the cytoplasm; consequently, CTE-containing HIV-1 RNAs were transported in the cytoplasm directionally and clustered to centrosomes that form the core of the microtubule-organizing center (MTOC). These studies suggested that the export pathway used by the RNA has a profound impact on the cytoplasmic trafficking and localization of RNA (33, 34).

To better understand how the nuclear export pathway affects HIV-1 RNA behavior, in this report, we compared the cytoplasmic trafficking of HIV-1 RNA containing the authentic RRE or the MPMV CTE in place of RRE. Using single particle tracking, we followed individual RNA molecules in the cytoplasm and found that the vast majority of the HIV-1 RNAs use diffusion as their transport mechanism. Interestingly, CTE-containing HIV-1 RNA diffuses at a lower rate than RRE-containing HIV-1 RNA. Using *in situ* hybridization, we analyzed cells dually infected with two proviruses, one containing CTE and the other containing RRE. Our results showed that CTE- and the RRE-containing HIV-1 RNAs have similar subcellular distributions. We measured the distances between individual RNAs to the centrosomes and found little if any difference between the CTE- and RRE-containing HIV-1 RNA. Thus, using live-cell imaging and *in situ* hybridization approaches, we observed that HIV-1 RNA exported through different pathways use similar RNA transport mechanisms and exhibit similar cytoplasmic distribution.

## RESULTS

### Detecting and tracking cytoplasmic HIV-1 RNA exported via distinct pathways.

**FIG 1:**
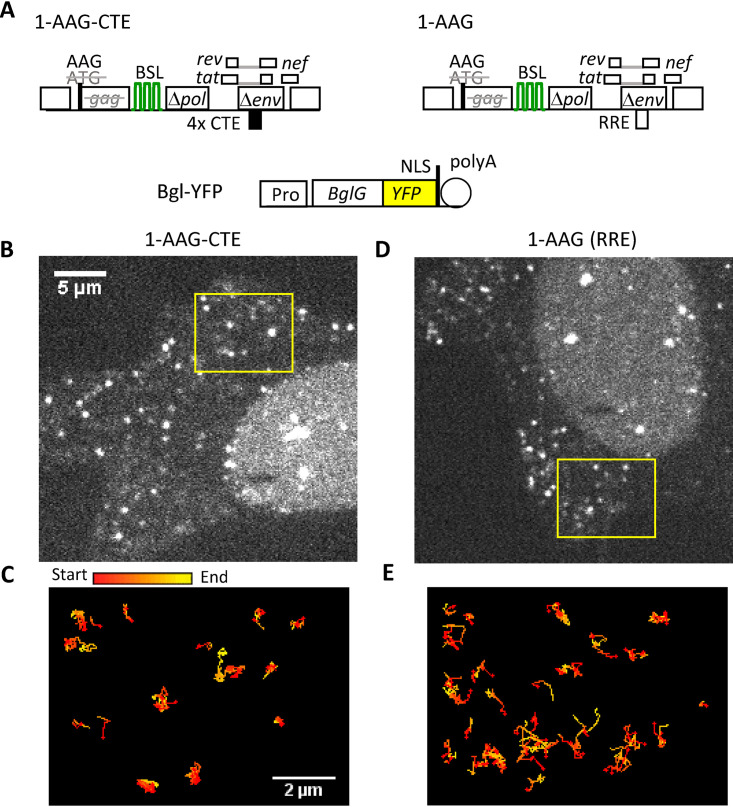
Visualizing cytoplasmic trafficking of HIV-1 RNA exported via different pathways. (A) General structures of the constructs expressing HIV-1 RNAs and RNA labeling protein. Both HIV-1 constructs contain an ATG to AAG mutation to abolish the expression of functional Gag. Black box labeled 4XCTE, four copies of CTE from MPMV; green stem loops, BSL sequences recognized by RNA-binding protein BglG. NLS, nuclear localization signal. (B to E) Representative images of cells expressing 1-AAG-CTE RNA (B) and 1-AAG RNA (D) are shown in the same scale; trajectories of RNA movement within 100 frames of the selected region indicated in (B) and (D) are shown in (C) and (E), respectively. Trajectories are depicted with the signals changing colors from start (red) to end (yellow). Panels C and E are shown at the same scale.

To examine the effects of nuclear export pathways on cytoplasmic RNA trafficking, we determined the mechanism of transport for HIV-1 RNA containing authentic RRE or replacing RRE with CTE. For this purpose, we generated an HIV-1 construct, 1-AAG-CTE, based on a previously described NL4-3-derived construct, 1-AAG (35) (Fig. 1A), by replacing the authentic RRE sequence with four copies of MPMV CTE. Therefore, 1-AAG and 1-AAG-CTE have similar structures, but their RNAs exit the nucleus using different pathways. Construct 1-AAG contains all the *cis*-acting elements necessary for viral replication and expresses Tat, Rev, and Nef. A T-to-A substitution of the *gag* translation start codon in 1-AAG and 1-AAG-CTE converts AUG to AAG, which abolishes Gag translation but does not interfere with RNA function, as the mutant RNA can be efficiently packaged into viral particles and undergoes one round of replication when essential proteins are provided in *trans* (35). These two constructs also contain a truncated *pol* gene in which 18 copies of stem-loop sequences (BSL) were inserted; BSL RNA is specifically recognized by the bacterial protein BglG (36, 37).

To visualize HIV-1 RNA, we transfected the 1-AAG-CTE construct into HeLa cells along with a plasmid expressing Bgl-yellow fluorescent protein (YFP) fusion protein (Fig. 1A), which binds to BSL-containing RNA (36, 37). The BSL is located in the *pol* gene of 1-AAG-CTE and is only present in unspliced RNA; thus, this system allows specific labeling of HIV-1 unspliced RNA. We visualized HIV-1 RNA in living cells using a spinning disk microscope imaging system and captured signals from the YFP channel at 42 ms per frame. As shown in representative images (Fig. 1B), multiple YFP puncta were detected in the cytoplasm; these signals were specific to Bgl-YFP-tagged HIV-1 RNAs because such puncta were not detected in the absence of Bgl-YFP or when HIV-1 RNAs lacked BSL. We observed that these RNA signals display dynamic motion in a nondirectional, random-walk manner (Movie S1). To better demonstrate the cytoplasmic HIV-1 RNA movement, we performed single particle tracking to follow individual RNAs shown in the box in Fig. 1B. The trajectories of these multiple RNAs during 100 frames are illustrated in Fig. 1C; these trajectories are color-coded; signals at the beginning of detection are shown as red, whereas the signals detected toward the end of the 100 frames are shown in yellow.

10.1128/mBio.01578-20.1 MOVIE S1 1-AAG-CTE RNA movement in the cytoplasm of a cell. HeLa cells were transfected with plasmids 1-AAG-CTE and Bgl-YFP (described in Fig. 1). Time-lapse images were acquired at 23.8 Hz with a 40-ms integration time. The movie is encoded at 24 Hz. Download Movie S1, AVI file, 8.6 MB. Copyright © 2020 Chen et al. 2020 Chen et al. This content is distributed under the terms of the Creative Commons Attribution 4.0 International license.

The behavior of the 1-AAG-CTE RNA observed was similar to the previously described 1-AAG RNA movement (31). For a direct comparison, we studied 1-AAG RNA using the same experimental conditions, including the imaging system. A representative image of 1-AAG YFP puncta in the cytoplasm is shown in Fig. 1D (also in the corresponding Movie S2); the movements of the individual RNAs are illustrated in Fig. 1E. These images showed that 1-AAG RNAs also move in a dynamic, random-walk manner, which is consistent with our previous studies (31).

10.1128/mBio.01578-20.2 MOVIE S2 1-AAG RNA movement in the cytoplasm of a cell. HeLa cells were transfected with plasmids 1-AAG and Bgl-YFP (described in Fig. 1). Time-lapse images were acquired at 23.8 Hz with a 40-ms integration time. The movie is encoded at 24 Hz. Download Movie S2, AVI file, 7.4 MB. Copyright © 2020 Chen et al. 2020 Chen et al. This content is distributed under the terms of the Creative Commons Attribution 4.0 International license.

### Comparing mobility and directionality of cytoplasmic HIV-1 RNA exported via different pathways.

**FIG 2:**
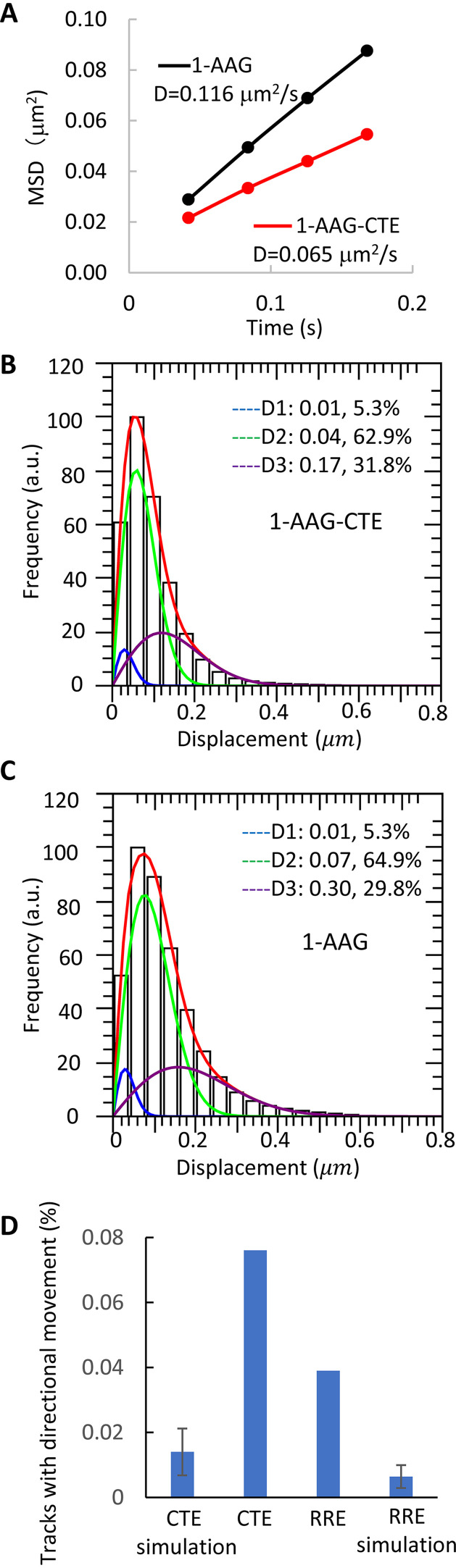
Mobility and directionality of cytoplasmic HIV-1 RNAs exported via different pathways. (A) Mean square displacement (MSD) analyses of 1-AAG-CTE (red) and 1-AAG (black) RNA. D, diffusion coefficient. (B and C) Distributions of the one-step jump distance of 1-AAG-CTE RNA (B) and 1-AAG RNA (C). Data were binned (40-nm bin size) and normalized to the bin that contained the most events, which was set to 100. *x* axis, one-step jump distance (displacement); *y* axis, frequency in arbitrary units (a.u.). The distributions were fitted with a three-component model using a constant diffusion coefficient (D1 = 0.01 μm^2^/s) to represent the stagnant fraction or mobility under the detection limit in our system. The solid red line represents the fitted curve, and the three dotted lines indicate distributions for each of the mobility fractions. The percentages shown are proportions of each fraction. (D) Proportion of RNA tracks with directional movement, which is defined as a segment that moved with a persistence index of ≥0.7 for ≥25 consecutive steps. Simulation was performed to generate 100 sets of random-walk tracks, with each set containing the same number of tracks, distribution of track length, and the same proportions of three diffusion coefficients based on results from 1-AAG-CTE or 1-AAG RNAs.

To better define the effects of the export pathway on HIV-1 RNA movement, we performed single-molecule tracking to follow 19,530 tracks of 1-AAG-CTE RNA from 52 cells and 35,766 tracks of 1-AAG RNA from 62 cells. We measured the movement of individual RNAs from one frame to the next, defined as a step. As most of the RNAs appeared to move in a nondirectional manner (Fig. 1), we measured the mean squared displacement (MSD) an RNA traveled within a given time. The ensemble MSDs for 1-AAG-CTE and 1-AAG RNA over four steps are shown in Fig. 2A. Results from both 1-AAG-CTE and 1-AAG showed a linear relationship between MSD and time (Fig. 2A), consistent with the observation that the majority of the RNAs move in a random-walk manner. Based on these results, we calculated the diffusion coefficient of these RNAs. Our results showed that the 1-AAG RNA diffused at a rate of 0.116 μm^2^/s, which is similar to what we previously measured using a wide-field microscope system (31). However, the 1-AAG-CTE RNA diffused at a rate of 0.065 μm^2^/s; which is close to half of the speed of the 1-AAG RNA.

The ensemble MSD analysis represents the average behavior of RNA tracks but does not distinguish whether there is more than one mobility behavior in the population. To better understand how the export pathway affects mobility of the cytoplasmic RNA, we calculated the jump distance traveled by each RNA track between two consecutive frames within 42 ms of imaging time (one-step jump distance). A total of 528,579 one-step jump distances were obtained from the 19,530 1-AAG-CTE RNA tracks, and 635,465 one-step jump distances were obtained from the 35,766 1-AAG RNA tracks. The distributions of these one-step jump distances are shown in Fig. 2B and C for 1-AAG-CTE and 1-AAG RNAs, respectively. The jump distance (displacement) is displayed on the *x* axis, and the frequency is displayed on the *y* axis, with the frequency of the distance that was most often traveled set to 100. The distribution of the one-step jump distances was heterogeneous and unlikely to fit the assumption of a single diffusion coefficient for 1-AAG-CTE and for 1-AAG-RNAs (*P* = 0.0000001 and *P = *0.000026, respectively, chi-square analysis). As we previously described (31), we obtained a satisfactory fit with the assumption that these RNAs could assume three mobility fractions: stagnant, intermediate, and fast. The probabilities that the fitting describe the RNA jump-distance distribution are *P = *0.94 for 1-AAG-CTE and *P = *0.97 for 1-AAG (chi-square analysis). The stagnant fraction is defined by the localization precision of our system, which was 40 nm, and yielded a diffusion coefficient of 0.01 μm^2^/s. Signals in the small stagnant fractions (blue lines in Fig. 2B and C) were either static or moved less than the localization precision of the system. The intermediate and fast fractions (green and purple lines, respectively, in Fig. 2B and C), exhibited mobility with coefficients of 0.04 μm^2^/s and 0.17 μm^2^/s, respectively, for 1-AAG-CTE RNAs, and with coefficients of 0.07 μm^2^/s and 0.30 μm^2^/s, respectively, for 1-AAG RNAs. These results are consistent with the MSD analysis that the 1-AAG-CTE RNAs diffuse at about half the rate of 1-AAG RNAs; however, both types of RNAs exhibited similar heterogenicity in mobility behavior.

To analyze the directionality of RNA molecules, we performed a previously described persistence index analysis (31), which examines whether an individual RNA travels toward the same direction in consecutive steps. It is possible for an RNA molecule to travel in the same direction for some steps during random-walk movement. To estimate the expected movement toward the same direction during random-walk movement, we used the measured mobility characteristics of the 19,530 1-AAG-CTE RNA tracks to perform simulation studies to model the behavior of random-walk movement. In 100 simulations, we found that ∼0.014% of the RNA tracks would contain a segment that exhibited a persistence index of ≥0.7 for 25 or more continuous steps, or an average of 2.8 tracks among the simulated 19,530 tracks for 1-AAG-CTE RNAs (Fig. 2D). We also performed simulations using the 1-AAG RNA mobility characteristics and found that ∼0.0064% of the RNA tracks would contain a segment that exhibited a persistence index of ≥0.7 for 25 or more continuous steps, or an average of 2.3 tracks among the 35,766 tracks in 100 simulations (Fig. 2D). Thus, we use these criteria to analyze the directionality of these two RNAs with our data sets. We found that 0.076% of the 1-AAG-CTE RNAs tracks (15 of the 19,530 tracks) and 0.039% of the 1-AAG RNAs tracks (14 of the 35,766 tracks) contained a segment with a persistence index of ≥0.7 for 25 or more continuous steps (Fig. 2D). These measured directional movements are more frequent than expected from random walks (*P = *0.0038 for 1-AAG-CTE and *P = *0.0037 for 1-AAG RNA, chi-squared test). However, 1-AAG-CTE RNAs do not move in a directional manner more frequently than 1-AAG RNAs (*P = *0.064, chi-squared test). Thus, the vast majority (>99%) of the HIV-1 RNAs use diffusion as a transport mechanism in the cytoplasm regardless of whether they were exported by the CRM1 or the NXF1 pathway.

### Effects of Gag expression on the cytoplasmic transport of HIV-1 RNA exported via the NXF1 pathway.

**FIG 3:**
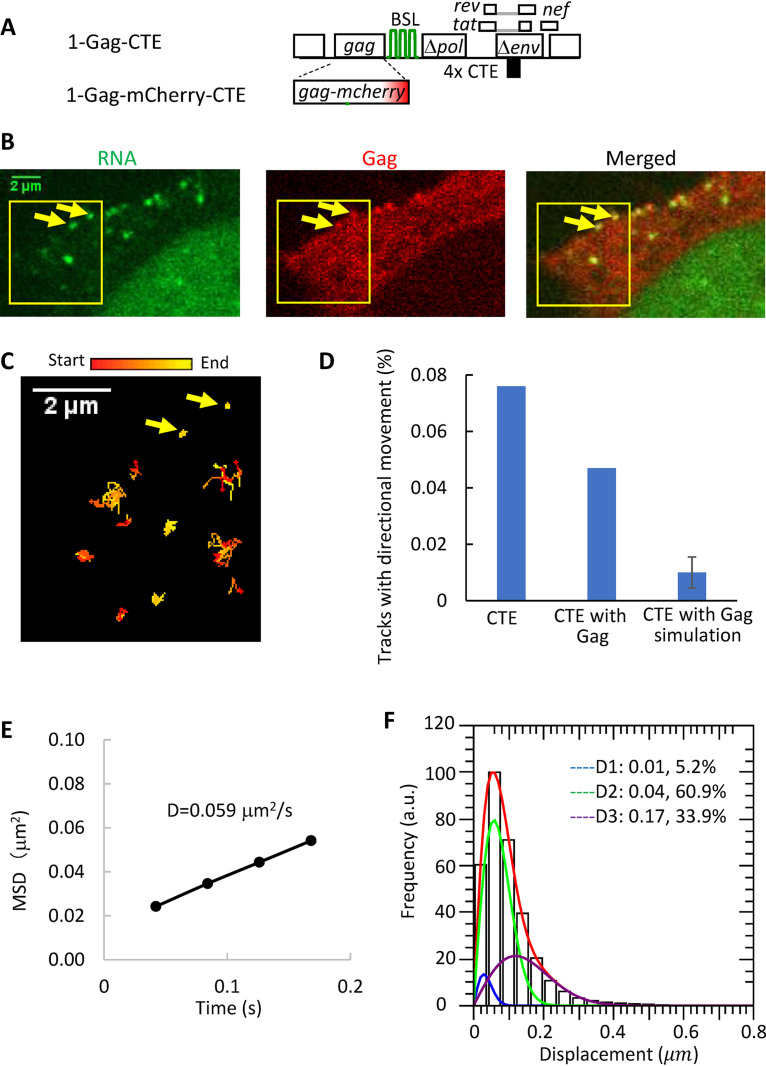
The effects of Gag on the transport of cytoplasmic HIV-1 RNA exported via the NXF1 pathway. (A) General structures of the HIV-1 constructs. (B) Representative images detected in YFP (RNA), mCherry (Gag), and both (merged) channels. Arrows indicate two colocalized RNA:Gag signals at the plasma membrane. An accompanying movie, Movie S3, shows 500 frames of the images. (C) Trajectories of HIV-1 RNA movement in the first 100 frames of the selected region (yellow boxes) indicated in panel B. Arrows indicate the same two signals as in panel B. Trajectories are depicted with changing colors from start (red) to end (yellow). (D) Persistence index analysis to detect tracks with directional movement. Simulation was performed based on tracking results of 1-Gag-CTE/1-Gag-mCherry-CTE. (E) MSD analysis of 1-Gag-CTE/1-GagmCherry-CTE RNA movement in the presence of Gag. D, diffusion coefficient. (F) Distribution of the one-step jump distance of 1-Gag-CTE/1-Gag-mcherry-CTE RNAs. The solid red line represents the fitted curve, and the three dotted lines indicate distributions for each of the mobility fractions. The percentages shown are proportions of each fraction.

The HIV-1 structural protein Gag is an RNA-binding protein, and its presence can potentially affect cytoplasmic RNA transport. To examine the potential effects of Gag on the cytoplasmic trafficking of CTE-containing HIV-1 RNA, we used two HIV-1 constructs that are structurally similar to 1-AAG-CTE but contain AUG at the Gag translational start codon; 1-Gag-CTE expresses an untagged Gag and 1-Gag-mCherry-CTE expresses an mCherry tagged Gag (Fig. 3A). We transfected 1-Gag-CTE, 1-Gag-mCherry-CTE, and Bgl-YFP into cells and monitored the appearance and movements of the cytoplasmic RNA and the Gag signals. We found that HIV-1 RNA signals first appeared in the cytoplasm before the detection of Gag puncta near the plasma membrane; furthermore, the numbers of both signals increased with time. These findings are similar to those observed with HIV-1 RNA containing authentic RRE and exported via the CRM1 pathway (31). To study the effects of Gag on cytoplasmic RNA movement, we selected cells with detectable Gag puncta at the plasma membrane, indicating the presence of sufficient amounts of Gag to support virion assembly (Fig. 3B). As shown in Fig. 3C and Movie S3, some of the RNAs were colocalized with the Gag puncta at the plasma membrane and lacked mobility, suggesting that these RNAs were part of the assembly complexes. In contrast, other RNAs were not colocalized with Gag puncta and moved dynamically in the cytoplasm; we followed the movements of these RNA signals and analyzed 25,352 RNA tracks from 44 cells. To analyze the distance individual RNAs traveled within a given time, we performed MSD analyses and found that the MSD value exhibits a linear relationship with time (R^2^ > 0.9), indicating that diffusion is the major transport mechanism. The diffusion coefficient calculated from the ensemble MSD values is 0.059 μm^2^/s (Fig. 3E), similar to that of 1-AAG-CTE (Fig. 2A) without the presence of Gag. We then analyzed the one-step jump distances of these RNA molecules; a total of 589,606 one-step jump distances were generated, which contained 5.2%, 60.9%, and 33.9% of stagnant, intermediate, and fast-moving populations, respectively; their distribution is shown in Fig. 3F. Using the mobility characteristics of the 25,352 RNA tracks, we modeled the behavior of random-walk movement and found that 0.01% of the RNA tracks was expected to have a persistence index of ≥0.7 for ≥25 consecutive steps during random-walk movement. We then analyzed the 25,352 tracks and found that 0.047% of the RNA tracks (12 of 25,352 tracks) contained a segment with a persistence index of ≥0.7 for 25 or more continuous steps (Fig. 3D), which is more frequent than expected from random-walk movement (*P = *0.01, chi-squared test) but similar to 1-AAG-CTE RNA (*P = *0.21, chi-squared test). Thus, similar to the movement of 1-AAG-CTE RNA in the absence of Gag, the vast majority of the tracks moved in a diffusive manner, and a very minor portion, less than 0.1%, moved in a directional manner. Taken together, the MSD, the one-step jump distance, and the persistence index analyses revealed that Gag expression does not have significant effects on the mobility or directionality of the cytoplasmic HIV-1 RNA exported via the NXF1 pathway. These results are reminiscent of our previous report wherein Gag did not affect the diffusion rate of cytoplasmic HIV-1 RNA containing authentic RRE that was exported by the CRM1 pathway.

10.1128/mBio.01578-20.3 MOVIE S3 HIV-1 RNA movement in the cytoplasm of a cell expressing functional Gag. HeLa cells were transfected with plasmids 1-GagmCherry-CTE/1-Gag-CTE (1:1 ratio; described in Fig. 3) and Bgl-YFP. Simultaneous two-channel, time-lapse images (RNA, green; Gag, red) were acquired at 23.8 Hz with a 40-ms integration time. The movie is encoded at 24 Hz. Download Movie S3, AVI file, 1.8 MB. Copyright © 2020 Chen et al. 2020 Chen et al. This content is distributed under the terms of the Creative Commons Attribution 4.0 International license.

### System to study the impact of export pathway on HIV-1 RNA expression kinetics and subcellular locations.

**FIG 4:**
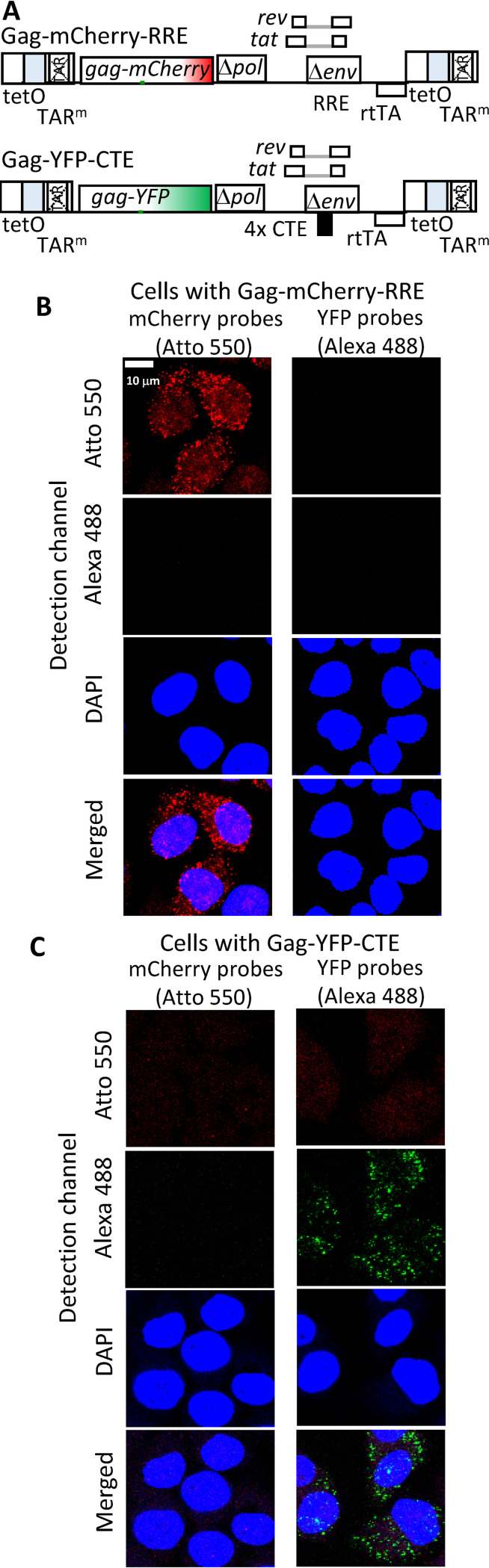
Experimental system used to study expression kinetics and cytoplasmic locations of HIV-1 RNA exported via the CRM1 or NXF1 pathway. (A) General structures of inducible HIV-1 constructs. In these constructs, the *rtTA* gene was inserted into the *nef* gene, and the tet operator (tetO) binding sites were inserted into the HIV-1 LTR. (B and C) Representative images of RNAscope detection of HIV-1 RNA derived from Gag-mCherry-RRE provirus (B) or Gag-YFP-CTE provirus (C). Probes specific to the *mCherry* or *yfp* gene used in the experiments are indicated above the panels. Channels used to detect signals are shown on the side of the images. Signals from *mCherry* probes are shown in red, whereas signals from *yfp* probes are shown in green. DAPI stains are shown in blue.

To directly compare the expression and cytoplasmic distribution of the HIV-1 RNA exported through different pathways, we established a cell line that expressed two proviruses, each using a different pathway to export its RNA. The general structures of these two constructs are shown in Fig. 4A; these constructs were modified from an engineered HIV-1 vector, HIV-rtTA, the expression of which is under doxycycline induction (38, 39). Briefly, in HIV-rtTA, inactivating mutations were introduced into *tat* and the Tat-binding site in the transactivation-response (TAR) element; the *rtTA* gene was inserted into the *nef* gene, and the tet operator (tetO) binding sites were inserted into HIV-1 LTR. Thus, the expression of HIV-rtTA is not regulated by viral protein Tat but can be induced by the addition of doxycycline. Importantly, HIV-rtTA generates a full-length RNA with an intact 5′ untranslated region, identical to wild-type HIV-1 except for several point mutations in TAR. HIV-rtTA was modified to generate Gag-mCherry-RRE, which expresses a Gag fused to mCherry fluorescent protein, Tat, and Rev and contains an authentic HIV-1 RRE. The construct Gag-YFP-CTE has a similar structure (Fig. 4A); however, it expresses a Gag-YFP fusion protein, and the RRE was replaced by four copies of MPMV CTE.

We generated a cell line that contained proviruses derived from Gag-mCherry-RRE and Gag-YFP-CTE by sequentially infecting HeLa cells at a low multiplicity of infection (MOI; <0.1). Infected cells were then enriched by repeated cell sorting until most of the cells (>90%) were dually positive with mCherry and YFP expression. Because cells were infected at low MOI, most of the infected cells contain only one of each provirus.

To detect HIV-1 RNA derived from Gag-mCherry-RRE and Gag-YFP-CTE, we used an *in situ* hybridization assay, RNAscope, to detect *mCherry* and *yfp* sequences, respectively. To determine whether this assay could distinguish between RNAs carrying *mCherry* or *yfp* sequences, we first tested cells infected with either Gag-mCherry-RRE or Gag-YFP-CTE. After the RNAscope procedure, we treated cells with DNA stain, DAPI (4′,6-diamidino-2-phenylindole), and captured images of cells using a confocal microscope in three channels to detect signals of probes targeting *mCherry* or *yfp* sequences and DAPI signals. A set of representative images of the equatorial planes is shown in Fig. 4B and C; for cells infected with Gag-mCherry-RRE, RNA signals were detected using *mCherry* probes but not *yfp* probes. Similarly, RNA signals were detected using *yfp* probes but not *mCherry* probes in cells infected with Gag-YFP-CTE. Thus, this assay can detect HIV-1 RNA derived from our constructs; furthermore, it can distinguish RNAs derived from Gag-mCherry-RRE and Gag-YFP-CTE.

### Comparing the expression kinetics and cytoplasmic locations of HIV-1 RNA exported using the CRM1 and NXF1 pathways.

**FIG 5:**
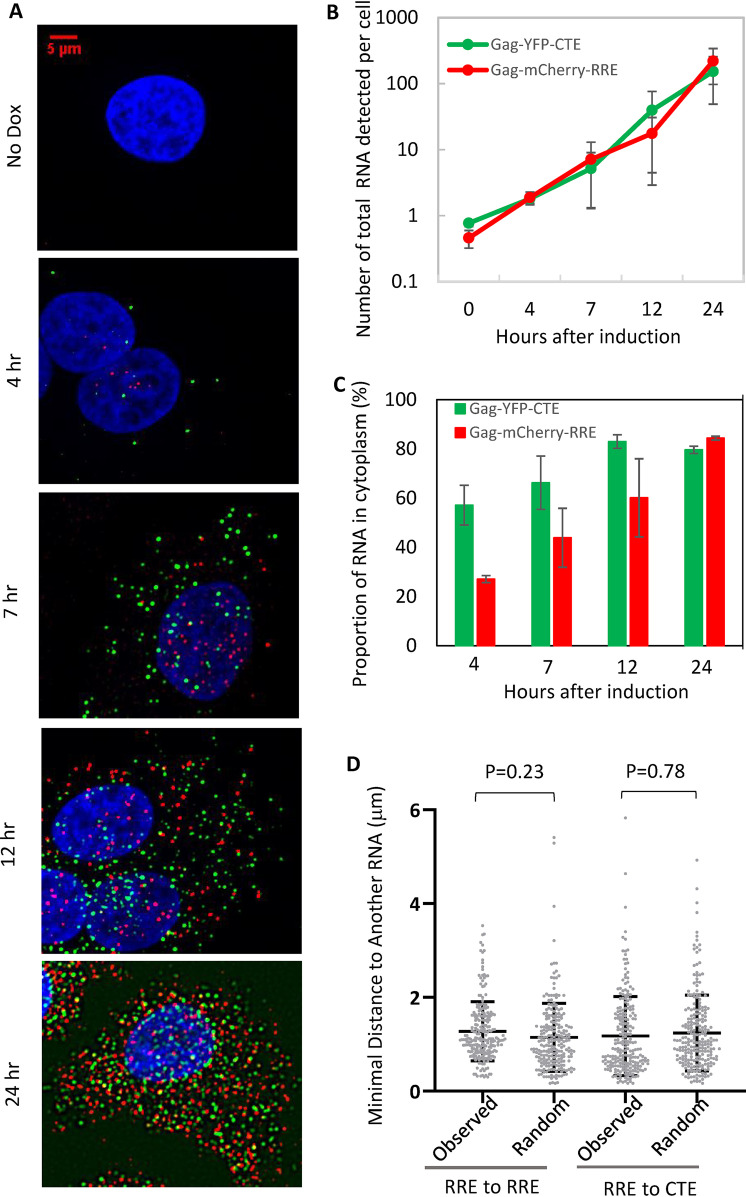
The expression kinetics and subcellular locations of HIV-1 RNA measured using a cell line dually infected with Gag-mCherry-RRE and Gag-YFP-CTE. (A) Representative images of HIV-1 RNA detected by RNAscope. Time points after doxycycline induction are shown to the left of the images. No dox, without doxycycline induction. Signals from *mCherry* probes, *yfp* probes, and DAPI stain are shown in red, green, and blue, respectively. (B) Quantitation of RNA accumulation after transcription induction. The number of total RNA signals detected in the equatorial plane of each cell is shown. The results shown are averages of 24, 45, 83, 56, and 34 cells for no dox (0 h) and 4 h, 7 h, 12 h, and 24 h after doxycycline induction, respectively. Error bars, standard deviation. (C) Quantitation of the proportion of RNA signals in the cytoplasm at the indicated hours postinduction. The proportion of cytoplasmic RNA is calculated by dividing the number of cytoplasmic RNAs by the number of total RNAs (RNA in cytoplasm and nucleus). (D) Spatial relationship between Gag-mCherry-RRE and Gag-YFP-CTE RNAs in the cytoplasm. The distances of individual Gag-mCherry-RRE RNA signals to the nearest Gag-mCherry-RRE RNA signal (RRE to RRE) or to the nearest Gag-YFP-CTE RNA signal (RRE to CTE) in the cytoplasm of a representative cell are shown. To generate distances expected from a cell in which Gag-mCherry-RRE and Gag-YFP-CTE RNAs were mixed randomly in the cytoplasm, we used the spatial information of Gag-mCherry-RRE and Gag-YFP-CTE RNAs. Based on the number of Gag-YFP-CTE molecules in the cytoplasm of the cell, we randomly assigned a subset of the total RNAs as Gag-YFP-CTE RNAs and measured the minimal distance of Gag-mCherry-RRE RNAs to Gag-mCherry-RRE RNAs or Gag-YFP-CTE RNAs; these values are shown as “random.”

To examine the kinetics of HIV-1 RNA expression, we treated the cells that were dually infected with Gag-mCherry-RRE and Gag-YFP-CTE with doxycycline, fixed cells at different time points, performed RNAscope, and captured images. Representative images are shown in Fig. 5A. Very few RNA signals were detected in the dually infected cells without doxycycline induction (no dox); after doxycycline induction, a few RNA signals could be detected at the 4 h time point, and the numbers of RNA signals increased with time and were abundant at the 24 h time point.

To quantify the expression kinetics, a series of z-stack images were collected for each field of view, and the images of the equatorial planes were selected for analyses. The number of RNA signals from the equatorial plane of each cell was determined, and results from 24, 45, 83, 56, and 34 cells for the no-dox (0 h), 4-h, 7-h, 12-h, and 24-h time points, respectively, are summarized in Fig. 5B. These results show that both CRM1-exported and NXF1-exported HIV-1 RNAs accumulate over time. The results in Fig. 5B represent the total number of RNAs detected in each cell; to distinguish between nuclear and cytoplasmic RNA, DAPI signals of each cell were used as a guide to generate a mask, and the numbers of cytoplasmic and nuclear RNAs were determined. Using these numbers, we calculated the proportion of RNA in the cytoplasm by dividing the number of cytoplasmic RNAs by total RNAs (cytoplasmic RNAs plus nuclear RNAs); these results are shown in Fig. 5C. The total numbers of RNAs from Gag-mCherry-RRE and Gag-YFP-CTE were similar at 4 h (Fig. 5B); however, the distributions of these RNAs differed. At the 4-h time point, ∼57% of the NXF1-exported Gag-YFP-CTE RNA was in the cytoplasm, whereas only ∼27% of the CRM1-exported Gag-mCherry-RRE RNA was in the cytoplasm. For both types of RNA, the proportion of cytoplasmic RNA increased with time, and by 24 h there were similar proportions of NXF1- and CRM1-exported HIV-1 RNA in the cytoplasm. These results indicate that both the NXF1 and CRM1 pathways export RNA efficiently; however, the export of the CRM1-dependent Gag-mCherry-RRE RNA is slower than that of the NXF1-dependent Gag-YFP-CTE RNA. These results are consistent with our understanding that a sufficient amount of Rev needs to be accumulated before RRE-containing HIV-1 RNA is exported (40) and with previous reports that CTE-containing RNAs are exported earlier than RRE-containing RNAs (33).

It has been suggested that the use of a particular RNA export pathway could affect the distribution of RNA in the cytoplasm (33). We reasoned that if RNAs exported by different pathways were located at distinct compartments in the cytoplasm, then the distances between RNAs exported using the same pathways should be shorter than that between RNAs exported using different pathways. To test this hypothesis, we measured the distances between each Gag-mCherry-RRE RNA to the nearest Gag-mCherry-RRE RNA (RRE to RRE), as well as distances between each Gag-mCherry-RRE RNA to the nearest Gag-YFP-CTE RNA (RRE to CTE). A set of representative results is shown in Fig. 5D as “observed.” We also used the spatial information of the RNA detected in the cytoplasm to perform a simulation assuming these two types of RNAs are randomly mixed and calculated the expected distances between RNAs (shown as “random” in Fig. 5D). Our results showed that the distances between Gag-mCherry-RRE RNA to the nearest Gag-mCherry-RRE RNA or Gag-YFP-CTE RNA were not different from those calculated based on random mixing of these two types of RNAs in a cell using simulation (RRE to RRE observed versus random, *P = *0.23; RRE to CTE observed versus random, *P = *0.78; one-way analysis of variance [ANOVA]). Additional data from analyses of two other cells are shown in Fig. S1.

10.1128/mBio.01578-20.4 FIG S1 The spatial relationship between Gag-mCherry-RRE and Gag-YFP-CTE RNAs in the cytoplasm of two cells in addition to Fig. 5D. Download FIG S1, TIF file, 1.4 MB. Copyright © 2020 Chen et al. 2020 Chen et al. This content is distributed under the terms of the Creative Commons Attribution 4.0 International license.

**FIG 6:**
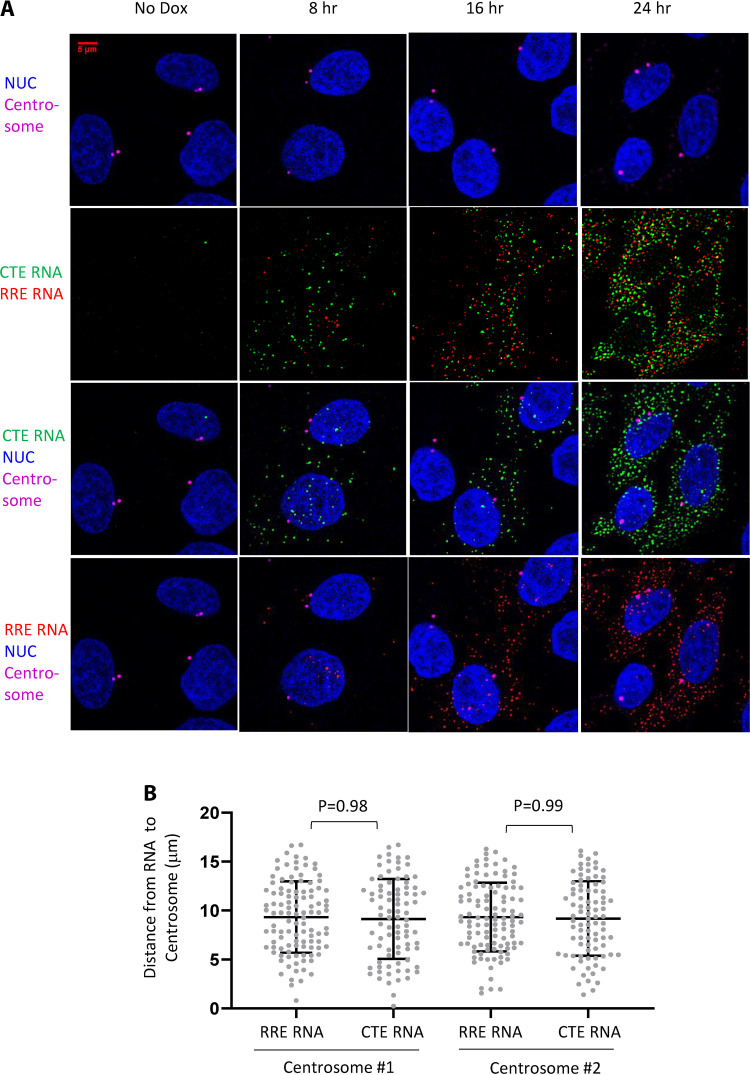
Distribution of HIV-1 RNAs relative to the centrosome. The expression of Gag-mCherry-RRE and Gag-YFP-CTE proviruses were induced by the addition of doxycycline, and cells were fixed at 8 h, 16 h, and 24 h postinduction. HIV-1 RNA was detected by RNAscope; Gag-YFP-CTE and Gag-mCherry-RRE RNA signals are shown in green and red, respectively. Centrosomes were detected by immunofluorescence with antibody to γ-tubulin. (A) Representative images showing HIV-1 RNA and centrosome signals. (B) The spatial relationship between Gag-mCherry RRE and Gag-YFP-CTE RNAs to centrosomes in the cytoplasm of the cell. The distances of individual Gag-mCherry-RRE and Gag-YFP-CTE RNAs to the two centrosomes detected from a representative cell are shown.

It was suggested that the MPMV CTE element links HIV-1 RNA to the microtubules in the cytoplasm, driving them to cluster at the centrosome (33). Analyses of our RNAscope images showed that HIV-1 RNAs exported through the NXF1 pathway were well mixed with RNAs exported through the CRM1 pathway in the cytoplasm. To further study the distribution of the HIV-1 RNAs, we combined immunofluorescence and RNAscope to detect centrosomes and HIV-1 RNA signals simultaneously in the same cell. We acquired four-channel z-stack images with a confocal microscope and analyzed the equatorial image plane with clearly identifiable centrosome signal(s). Our images did not reveal accumulation or clustering of Gag-YFP-CTE RNAs or Gag-mCherry-RRE RNAs at the centrosomes in cells (Fig. 6A). To quantify the spatial relationship of the HIV-1 RNAs to the centrosomes, we measured the distances of all Gag-mCherry-RRE RNAs and Gag-YFP-CTE RNAs to the two centrosomes in the cell and observed similar distance distributions of Gag-mCherry-RRE RNAs and Gag-YFP-CTE RNAs to both centrosomes (Fig. 6B; centrosome 1, Gag-mCherry-RRE RNA versus Gag-YFP-CTE RNA, *P = *0.98; centrosome 2, Gag-mCherry-RRE RNA versus Gag-YFP-CTE RNA, *P = *0.99; one-way ANOVA). Results from analyses of 27 additional cells are shown in Table 1. In the analyzed image plane, both centrosomes were visible in 17 of the 27 cells, and only one centrosome was visible in the equatorial plane in the other 10 cells. Along with the cell shown in Fig. 6, we compared the distances of HIV-1 RNAs containing CTE or RRE to centrosomes in 28 cells and 46 centrosomes. In all cases, the average distance of the Gag-YFP-CTE RNAs to a centrosome is not significantly different than the distance of the Gag-mCherry-RRE RNAs to the same centrosome. Thus, our results show that RRE- and CTE-containing HIV-1 RNAs have similar subcellular distributions and do not display differences in their localization relative to the centrosomes.

**TABLE 1:** Distances of CTE RNA and RRE RNA to centrosomes^*a*^

Cell no.	Distance of centrosome 1 to (μm):			Distance of centrosome 2 to (μm):		
	CTE RNA (avg ± SD)	RRE RNA (avg ± SD)	*P*	CTE RNA (avg ± SD)	RRE RNA (avg ± SD)	*P*
1	8.47 ± 3.14	8.58 ± 3.56	0.79	NA	NA	NA
2	10.06 ± 3.70	10.22 ± 3.60	0.64	10.06 ± 3.76	10.18 ± 3.67	0.73
3	9.89 ± 4.72	9.94 ± 10.01	0.51	11.05 ± 8.35	10.85 ± 11.04	0.37
4	14.50 ± 6.22	16.08 ± 5.69	0.10	NA	NA	NA
5	8.57 ± 3.22	8.21 ± 3.14	0.45	8.86 ± 4.47	8.15 ± 4.13	0.27
6	7.70 ± 3.78	7.56 ± 3.41	0.72	8.06 ± 3.28	8.22 ± 3.06	0.63
7	8.93 ± 3.17	9.22 ± 3.23	0.44	8.59 ± 3.71	8.96 ± 3.86	0.40
8	7.02 ± 2.72	7.09 ± 2.49	0.85	6.82 ± 2.75	6.80 ± 2.56	0.94
9	12.27 ± 6.08	13.28 ± 6.18	0.17	9.13 ± 3.22	9.40 ± 3.44	0.49
10	8.56 ± 4.38	8.56 ± 4.39	0.99	8.42 ± 3.63	8.87 ± 3.34	0.16
11	10.82 ± 3.41	11.05 ± 3.46	0.48	11.26 ± 3.66	11.37 ± 3.64	0.77
12	13.42 ± 5.40	14.09 ± 4.90	0.10	13.68 ± 6.19	14.15 ± 5.69	0.32
13	10.04 ± 4.15	9.86 ± 4.16	0.61	11.14 ± 4.10	11.42 ± 4.15	0.42
14	11.00 ± 5.89	11.13 ± 5.72	0.84	NA	NA	NA
15	11.24 ± 4.38	12.08 ± 4.62	0.07	NA	NA	NA
16	7.89 ± 3.10	7.92 ± 3.43	0.94	7.87 ± 3.98	7.88 ± 4.37	0.99
17	5.88 ± 2.97	5.31 ± 2.91	0.32	NA	NA	NA
18	9.84 ± 9.40	10.17 ± 2.98	0.48	NA	NA	NA
19	9.63 ± 4.53	9.62 ± 4.36	0.99	9.65 ± 4.86	9.62 ± 4.36	0.96
20	9.43 ± 3.15	9.68 ± 3.16	0.46	NA	NA	NA
21	11.23 ± 5.85	12.00 ± 5.86	0.34	NA	NA	NA
22	9.42 ± 2.78	9.14 ± 3.02	0.43	9.90 ± 4.05	9.67 ± 4.35	0.65
23	11.18 ± 5.66	10.68 ± 5.88	0.48	10.39 ± 4.36	9.81 ± 4.63	0.29
24	9.50 ± 4.23	9.92 ± 4.44	0.45	NA	NA	NA
25	7.03 ± 3.83	7.23 ± 3.78	0.63	7.12 ± 3.45	7.35 ± 3.38	0.55
26	14.16 ± 5.00	13.72 ± 5.41	0.41	7.68 ± 3.87	7.86 ± 3.52	0.64
27	7.24 ± 4.00	7.67 ± 3.86	0.40	NA	NA	NA

a NA, only one centrosome detected at the equatorial plane in those cells.

Taken together, these results indicate that the vast majority of the HIV-1 RNA exported by CRM1- or NXF1-mediated pathways use diffusion to travel in the cytoplasm. However, the export pathways used by HIV-1 RNAs affect the mobility of the RNAs in the cytoplasm; HIV-1 RNA using the NXF1 export pathway diffuses at a lower rate than its counterpart exported by the CRM1 pathway. HIV-1 RNAs are exported efficiently by both CRM1 and NXF1 pathways, although RNAs exported through the CRM1 pathway accumulate in the cytoplasm more slowly than those exported through the NXF1 pathway, most likely because sufficient Rev expression is needed for the RNA transport. HIV-1 RNAs exported via the CRM1 or the NXF1 pathway have similar cytoplasmic distributions and do not cluster near the centrosomes.

## DISCUSSION

HIV-1 RNA must be exported into the cytoplasm to carry out its functions. Proper nuclear export not only allows HIV-1 RNA to localize in the cytoplasm, but also affects RNA functions and downstream events in viral replication. It has been shown that although HIV-1 RNA lacking RRE or Rev can be detected in the cytoplasm, it was not efficiently packaged or translated (41–43). Similarly, HIV-1 RNA is not properly exported in murine cells because the mouse CRM1 protein does not efficiently multimerize on the HIV-1 Rev/RRE complex; this defect not only resulted in inefficient export, but also partly contributed to the observed Gag assembly defects in murine cells (20, 44, 45). These defects can be rescued by the presence of CTE in the viral RNA; CTE-containing HIV-1 RNA can be packaged into particles and efficiently translated to correct HIV-1 assembly defects in murine cells (20, 41, 46). Thus, CTE can functionally replace the requirement for Rev/RRE in HIV-1 RNA. However, it was unclear whether the physical properties of the HIV-1 RNA exported using these two pathways were different; CTE-containing HIV-1 RNA was suggested to use a different mode of transport and have distinct cytoplasmic locations (33). In this report, we compared the movements of CTE- and RRE-containing HIV-1 RNAs using single-molecule tracking and showed that both RNAs mainly use diffusion to travel in the cytoplasm. We also used RNAscope to identify RRE- or CTE-containing HIV-1 RNA in the same cell and compared their subcellular locations. Our studies show that these two types of RNAs are well mixed in the cytoplasm and have similar subcellular distributions.

To compare the movements of the RRE- and CTE-containing HIV-1 RNAs, we performed single-molecule tracking to follow a large number of RNA tracks. We then examined whether individual RNAs traveled in the same direction in consecutive steps using persistence index analyses. We found that >99% of the CTE- and RRE-containing HIV-1 RNA travel in a random-walk manner; furthermore, the proportion of CTE-containing RNA engaging in directional movement (0.076%) is not significantly different than that of RRE-containing RNA (0.039%). These results are different than the previous report indicating that CTE-containing HIV-1 RNA was transported in a directional manner (33). At this time, the reasons behind the observed differences are unclear. One speculation is that the speed at which the RNA images were captured could affect the ability to track RNA and influence data interpretation. HIV-1 RNAs move dynamically in the cytoplasm, and we used high-speed imaging to capture their movements (42 ms per frames or ∼24 frames per second). If the images of the RNAs were captured with a long lag time between frames, such an approach could result in difficulty tracking diffusive movement, thereby elevating the ratios of the observed directional movement.

Although we observed that both CTE- and RRE-containing RNAs use diffusion to travel within the cytoplasm, we observed that these two types of RNAs have different diffusion rates. The diffusion rate of 1-AAG RNA (0.116 μm^2^/s) is faster than the rate of 1-AAG-CTE RNA (0.065 μm^2^/s). These results were generated from measuring the distances traveled by these two types of RNAs in four steps and used the results to calculate ensemble MSDs (Fig. 2A). We compared the results in each of the four steps between these 1-AAG and 1-AAG-CTE and found that each of the four data points is significantly different (*P* < 10^−6^, two-tailed unequal variance *t* test). The relationship between mass and the rate of diffusion is cubed, suggesting that the mass of the CTE-containing RNA is significantly larger than that of the RRE-containing HIV-1 RNA. However, 1-AAG-CTE RNA is only 78 nucleotides (nt), or 1.5%, longer than the 1-AAG RNA; thus, the difference in diffusion rates cannot be attributed to the difference in length between the two RNAs. Most of the cellular mRNAs are associated with proteins to form ribonucleoprotein (RNP) complexes. It is possible that the CTE- and RRE-containing HIV-1 RNAs have distinct protein compositions; many aspects of the proteins associated with cytoplasmic RNAs are undefined, including when the NXF1/NXT1 complexes dissociate from the exported HIV-1 RNA. Alternatively, it is also possible that HIV-1 RNAs exported through NXF1 pathways have distinct RNA folding and structures from RNA exported via CRM1 pathway, resulting in the difference in diffusion rates. Future experiments will be needed to identify the reasons for the differences in diffusion rates.

We have also compared the subcellular location of CTE- and RRE-containing HIV-1 RNAs using a cell line containing two doxycycline inducible proviruses. Using RNAscope, we compared CTE- and RRE-containing HIV-1 RNAs in the same cells. In these analyses, we examined whether RNAs exported through the same pathways cluster together and whether the CTE- and RRE-containing RNAs have different distances to a centrosome. In these analyses we observed that CTE- and RRE-containing HIV-1 RNAs are well mixed in the cytoplasm. It was reported that in ∼25% of the cells, CTE-containing RNAs were clustered near centrosomes (33). We analyzed 28 cells and did not observe clustering of CTE-containing RNA near centrosomes; this is significantly different than expected from 25% of the cells exhibiting such a phenotype (*P = *0.0047; chi-square analyses with the assumption that 7 cells [25% of 28 cells] display clustering). Thus, HIV-1 RNAs do not cluster near centrosomes in an export pathway-dependent manner. These results are distinct from the previous observations (33). At this time, it is unclear why the two studies have distinct findings; we speculate that experimental systems used in the studies may contribute to the observed differences. In our studies described in this report, HIV-1 RNAs were expressed from proviruses, whereas in the previous study, HIV-1 RNAs were expressed from transfected DNAs. It is possible that transfection resulted in RNA expression at levels higher than that from a provirus and caused the observed differences. Alternatively, other distinct biological features in the two experimental systems could have caused the observed differences.

We previously observed that HIV-1 RNAs containing CTE or RRE can both be packaged into viral particles; however, these two types of RNA do not copackage together efficiently (22). To explain this observation, we hypothesized that CTE- and RRE-containing HIV-1 RNAs are segregated in the cytoplasm (22). Here, we showed that CTE- and RRE-containing RNAs are well-mixed in the cytoplasm. Together with our observation that HIV-1 RNA dimerization occurs at the plasma membrane, these results suggest that the inefficient copackaging of the CTE- and RRE-containing RNAs is not caused by spatial segregation of RNA in the cytoplasm. Further studies will be needed to decipher the mechanisms by which export pathways affect RNA copackaging.

We also examined the expression and export kinetics of the RRE- and CTE-containing HIV-1 RNA. The probes we used to detect *mCherry* or *yfp* sequences allow us to only observe full-length unspliced RNAs. We observed that, after doxycycline induction, there are similar total numbers of CTE- and RRE-containing full-length RNAs between 4 to 24 h (Fig. 5B). However, the export of the RRE-containing HIV-1 RNA is slower than that of the CTE-containing HIV-1 RNA (Fig. 5C). For the CTE-containing RNA, the proportion of RNA increases in the cytoplasm with time; however, even at an early time point, such as 4 h, ∼60% of the RNA is in the cytoplasm. In contrast, the majority of RRE-containing HIV-1 RNAs are in the nucleus at 4 h, but the ratios of the cytoplasmic RNAs increase with time, and by 24 h, most of the RRE-containing HIV-1 RNAs are in the cytoplasm. The slower export kinetics of the RRE-containing HIV-1 RNA is consistent with the requirement for Rev expression, which is translated from a completely spliced RNA.

The nuclear export of HIV-1 RNA is important for its functions both as an encapsidated virion genome and as a template for Gag/Gag-Pol translation. We have shown in this report that whether RNA export is mediated by the CRM1 or the NXF1 pathway, HIV-1 cytoplasmic RNA mainly travels by diffusion and results in similar distribution patterns. These studies underscore the flexible nature of HIV-1 replication and the ability of the virus to adapt.

## MATERIALS AND METHODS

### Plasmids.

Plasmids 1-AAG and Bgl-YFP have been described previously (31, 35). Plasmid 1-AAG-CTE was generated by replacing the SalI to NgoMIV fragment of 1-AAG with the corresponding fragment of GagCeFP-BglSL-CTE (22). Plasmid 1-GagmCherry-CTE was generated by replacing the SalI/NgoMIV fragment of 1-GagmCherry-BSL (31) with the corresponding fragment of 1-AAG-CTE. Doxycycline-inducible HIV-1 plasmids bearing RRE or CTE export signals were derived from HIV-rtTA (38, 39). Briefly, to obtain Gag-mCherry-RRE, a large portion of the viral sequence (fragments SfoI to AgeI) including the *gag-pol* sequence in HIV-rtTA was replaced by the counterparts (fragments SfoI to Agel) from 1-GagmCherry-BSL (31). To obtain Gag-YFP-CTE, the mCherry sequence in Gag-mCherry-RRE was replaced with a YFP sequence using XbaI/BsrG1, and the RRE sequence was replaced with a CTE sequence from GagCeFP-BglSL-CTE (22) using AscI/SanDI sites. Molecular cloning was performed using standard techniques; the general structures of all plasmids were verified by restriction enzyme mapping, and DNA sequences generated by PCR were confirmed by DNA sequencing to avoid inadvertent mutations.

### Cell culture, transfection, and generation of provirus-expressing cell lines.

Human 293T cells and HeLa cells were maintained in Dulbecco’s modified Eagle’s medium (DMEM) supplemented with 10% fetal bovine serum (HyClone), penicillin (50 U/ml; Gibco), and streptomycin (50 μg/ml; Gibco), and cells were maintained in humidified 37°C incubators with 5% CO_2_.

Transient transfections were performed using FuGENE HD transfection reagent (Promega) according to the manufacturer’s recommendations. HeLa cell lines that contain Gag-mCherry-RRE and Gag-YFP-CTE proviruses were generated by sequential infection and cell sorting. Briefly, an HIV-1 construct was cotransfected into 293T cells with pC-help and pHCMV-G plasmids, which express HIV-1 proteins (47) and vesicular stomatitis virus G protein (48), respectively. Viruses were harvested from transfected cells 24 h later, clarified through a 0.45-μm-pore-size filter to remove cellular debris, and used for infection. HeLa cells were sequentially infected at low MOI (<0.1) to ensure that most infected cells only contained one copy of each provirus. The dually infected cell pool contained at least 6,000 independently infected cells and was enriched by repeated cell sorting until >80% of the cells in the population expressed Gag-mCherry-RRE and Gag-YFP-CTE. Proviral expression was induced by the addition of 3 μg/ml doxycycline (final concentration) to the cell culture.

### RNAscope fluorescent multiplex and immunofluorescent assay.

An RNAscope assay was performed according to the manufacturer’s recommendation (ACDbio) with probes specific to the *mCherry* gene (RNAscope Probe-mCherry-C2; product number 431201-C2) or the *yfp* gene (RNAscope Probe-EYFP; product 312131). To detect centrosomes, following the RNAscope procedure, cells were treated with phosphate-buffered saline with 3% bovine serum albumin for 45 min at room temperature, followed by incubation with a mouse antibody against human γ-tubulin (Sigma) for 2 h at room temperature. After washing with phosphate-buffered saline containing 0.2% Tween 20, cells were incubated with Cy5-tagged anti-mouse antibody for 30 min at room temperature and washed, and this was followed by DAPI stain for 5 min. The cells were then mounted with mounting solution and sealed for imaging. Images of 3 or 4 channels to detect DAPI, Alexa 488 (for *yfp* sequence detection), Atto 550 (for *mCherry* sequence detection), and Cy5 (centrosome) were acquired with a Zeiss Lsm780 confocal microscope. A customized Matlab program was used to select the equatorial plane from acquired z-stack images based on the area of nuclear DAPI stain. Gag-mCherry-RRE and Gag-YFP-CTE RNA signals were identified with the Localize program (49). A customized Matlab program was used to sort the Gag-mCherry-RRE and Gag-YFP-CTE RNA signals located in the nucleus and cytoplasm based on nuclear masks generated from DAPI staining.

### Microscopy, imaging acquisition, processing, and data analysis.

For live-cell imaging, spinning disk confocal microscopy was performed using an inverted Nikon Ti microscope with the Yokogawa CSU-X1 confocal scanner unit and a 100 × 1.45-numerical aperture (100 × 1.45-NA) total internal-reflection fluorescence (TIRF) oil objective. Simultaneous imaging of YFP and mCherry was performed by using two precisely aligned cameras (Andor iXon Ultra) on a Cairn image splitter (Optosplit II). Camera alignments were performed using labeled HIV-1 particles as previously described (31). The YFP and mCherry were excited with 514- and 594-nm lasers, respectively, whereas emission was detected by using 542/27- and 650/75-nm filters, respectively. Rapid HIV-1 RNA movement was acquired by using RAM capture with a 40-ms integration time and ∼2-ms overhead between frames, resulting in an overall 42-ms frame time. Subsequent image processing and analyses, including Laplacian of Gaussian (LoG) filtering and movie encoding, were performed with ImageJ software.

### Single-molecule tracking and analysis.

Single-molecule tracking was performed with Matlab (Mathworks) code (http://physics.georgetown.edu/matlab/) based on the available tracking algorithms (50) with maximum single-step displacement of 4 pixels (0.52 μm) and minimum track length of 5 consecutive frames. The positions of the diffraction-limited spots in the trajectories were refined with two-dimensional (2D) Gaussian fitting (51). MSDs were calculated from positional coordinates as previously described (52). In free diffusion, the MSDs [*r*^2^(*t*)], are linearly related to time (*t*) and the diffusion coefficient (D) by the formula *r*^2^(*t*) = 4D*t*.

In jump-distance analysis, the probability that a particle starting at a specific position will be encountered within a shell of radius (*r*) and width (*dr*) at time (*t*) from that position is given as $p(r,t)\mathrm{dt}=\frac{1}{4\pi \mathrm{Dt}}e^{-r^{2}/4Dt}2\pi \mathrm{dr}$ when starting at the origin. Experimentally, this probability distribution can be approximated by a frequency distribution, which is obtained by counting the jump distances within respective intervals (*r*, *r* + *dr*) traveled by a single RNA during a given time. When a population contains multiple diffusive fractions, the jump-distance distribution cannot be fitted satisfactorily by the above function with a single diffusion coefficient. Such different mobility fractions can be detected and quantified by curve fitting, taking several diffusion terms into account (53).

The persistence index of a track between two points was calculated by dividing the net distance of the track between two points by the total distance, using the formula $P_{\mathrm{index}}=\frac{d\left(p1,\mathrm{pL}\right)}{\sum_{i=1}^{L-1}d\left(p_{i},p_{i+1}\right)}$, where *d* represents the distance, *p*1 represents the initial point, and *pL* represents the last point. A computer program based on Matlab was generated to simulate free diffusion in 2D. Using this program, and based on Gaussian random-walk movement, we generated 100 sets of simulation with the same number and length distribution of trajectories and proportion of the three diffusion coefficients as the 1-AAG, 1-AAG-CTE, and 1-GagmCherry-CTE data sets, respectively. To identify potential directional mobility, we scanned the experimental trajectories and 100 sets of simulated trajectories for segments consisting of 10 to 48 consecutive steps with a persistence index ≥ 0.7.
