# Supplementary figures and images for: Impact of Nuclear Export Pathway on Cytoplasmic HIV-1 RNA Transport Mechanism and Distribution
Source: mBio. 2020 Nov 10;11(6):e01578-20. doi: 10.1128/mBio.01578-20 (PMC7667035; doi:10.1128/mBio.01578-20)

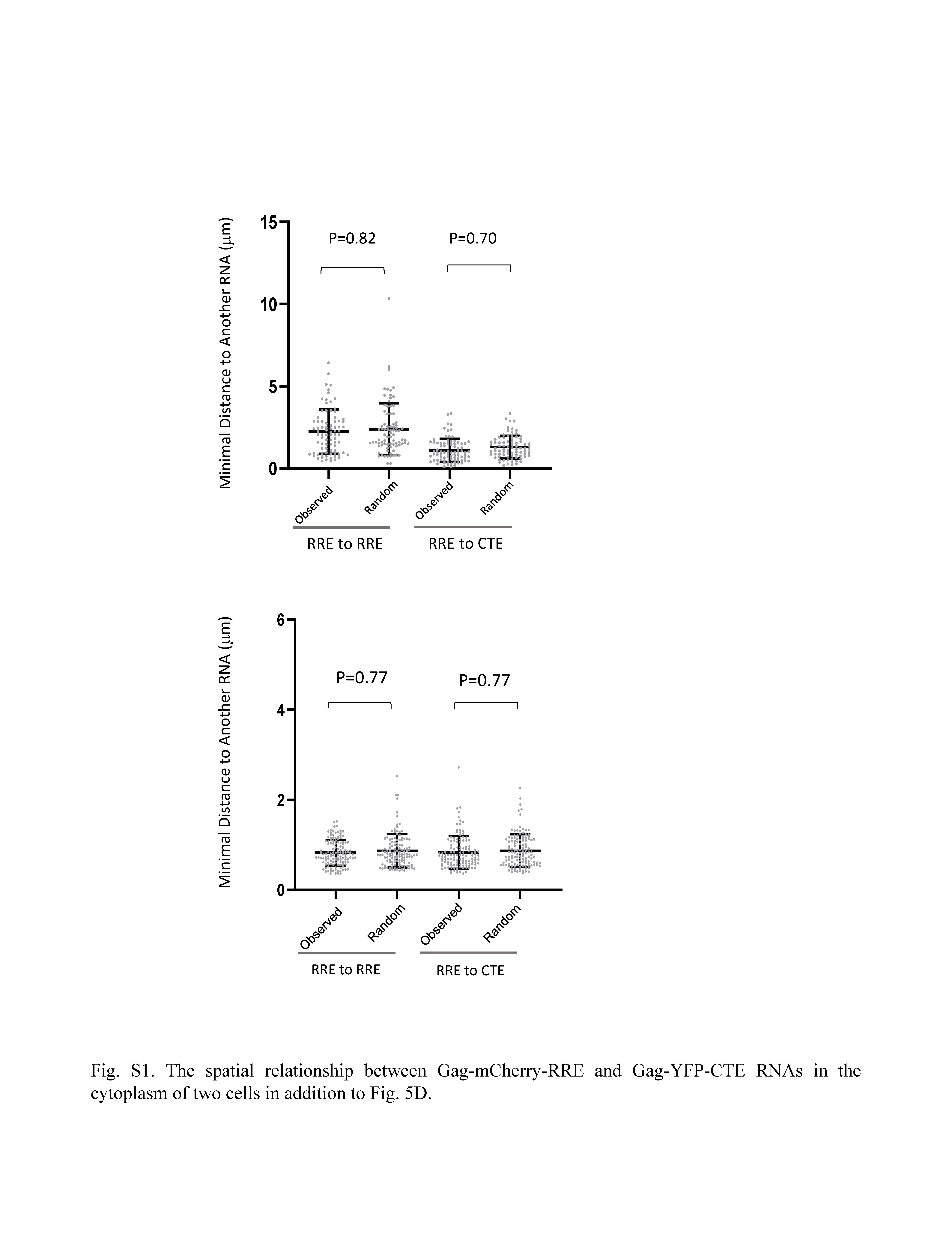

Supplement: FIG S1 [file mBio.01578-20-sf001.tif]
